# Hypocholesterolaemic and Anti-Atherogenic Effects of Palm-Based Oils (NoveLin I and NoveLin II) in Cholesterol-Fed Rabbits

**DOI:** 10.3390/ijerph17093226

**Published:** 2020-05-06

**Authors:** Che Anishas Che Idris, Siew Wai Lin, Ahmad Faizal Abdull Razis

**Affiliations:** 1 Malaysian Palm Oil Board, No. 6, Persiaran Institusi, Bandar Baru Bangi, Kajang 43000, Selangor, Malaysia; anis@mpob.gov.my (C.A.C.I.); wailin28@gmail.com (S.W.L.); 2 Laboratory of Molecular Biomedicine, Institute of Bioscience, Universiti Putra Malaysia, Serdang 43400 UPM, Selangor, Malaysia; 3 Laboratory of Food Safety and Food Integrity, Institute of Tropical Agriculture and Food Security, Universiti Putra Malaysia, Serdang 43400 UPM, Selangor, Malaysia; 4 Faculty of Food Science and Technology, Universiti Putra Malaysia, Serdang 43400 UPM, Selangor, Malaysia

**Keywords:** NoveLin I, NoveLin II, palm-based, cholesterol, atherosclerosis

## Abstract

NoveLin I and NoveLin II are palm-based oils. NoveLin I has an equal distribution of saturated, monounsaturated and polyunsaturated fatty acids, whereas NoveLin II has a moderate level of monounsaturated fatty acids, and a lower content of saturated and polyunsaturated fatty acids. However, their hypocholesterolaemic and anti-atherogenic effects have not been studied. Therefore, this study aimed to assess the hypocholesterolaemic and anti-atherogenic effects of these oils. Forty male New Zealand White rabbits were divided into four groups and fed with diets containing 35% energy fat with added 0.15% (w/w) dietary cholesterol. Group 1, as the control group (CNO) was fed with a diet containing coconut oil, group 2 and 3 were fed with diets containing either NoveLin I or NoveLin II, and group 4, was fed with diet containing olive oil (OLV) for 100 days. Our results demonstrated that both NoveLin groups have significantly lower total cholesterol and low-density lipoprotein–cholesterol (LDL–C) compared to CNO group and are comparable to the OLV group. Low density lipoprotein–cholesterol/high density lipoprotein-cholesterol (LDL/HDL–C) ratio was significantly lower after the NoveLin II diet but attained significance only in comparison to NoveLin I and CNO groups. Aortic fibrous plaque score was significantly lower in both NoveLin groups compared to CNO group. Our findings suggest that despite the high-fat cholesterol diet, NoveLin II oil resulted in atherogenic effects comparable to olive oil.

## 1. Introduction

Atherosclerosis is the main underlying cause of heart disease, which is the mutual cause of death in developed countries [1,2]. The atherosclerosis process initiates as a protective response to injuries to the endothelium and smooth muscle cells of the arterial wall and leads to atherosclerotic plaque formation [2]. This in turn narrows, and may block, the lumen of the affected artery. Atherosclerosis is responsible for myocardial ischemia/infarction, stroke, aortic aneurysms and peripheral vascular diseases [2].

The relationship among diet, plasma lipid concentrations and atherosclerosis has been well documented and reviewed [3]. Atherosclerosis lesions in animals and humans seem to be associated with elevated plasma total cholesterol (TC), decreased high-density lipoprotein cholesterol (HDL–C), increased low-density lipoprotein cholesterol (LDL–C) and over-consumption of fat [4]. Dietary fats can be important determinants that regulate blood cholesterol levels. Generally, saturated fats are thought to increase blood cholesterol whereas unsaturated fats decrease blood cholesterol. Chiu et al. [5] reported that unsaturated fats were less atherogenic than saturated fats and this outcome was further reduced by a cholesterol-poor diet.

Palm oil having 50% of its fatty acid composition as saturated fatty acids has been postulated to increase coronary heart disease risk by elevating blood cholesterol levels [6]. However, several human clinical trials, including those from our laboratories, have discounted this postulation [4,7]. Instead it has been shown that palm olein could be used to replace canola, olive and rapeseed oils without affecting lipoprotein cholesterol and serum lipids levels [8,9]. In human studies designed to evaluate the atherosclerotic effects of palm-based diets, it was noted that the effects of palm oil were unlike saturated fats but resembled those of unsaturated fats [10]. The existence of numerous fat-soluble micronutrients in palm oil, namely vitamin E, carotenoids and phytosterols, contributes to its pleiotropic nutritious profile. Oil palm vitamin E has been reported to act as a potent biological antioxidant which was suggested to reduce cardiovascular disease by modulating human lipids and lipoproteins [6].

The American Heart Association (AHA) Nutrition Committee (2006) [11] recommended that the fats people eat should have equal parts of saturated, monounsaturated and polyunsaturated fatty acids (Step I diet). This should have a distribution ratio 1:1:1 of all the fatty acids but in nature, no oils or fats that is available for consumption have this recommended ratio.

Previously, Sundram et al. [7,12] designed an AHA oil blend which give rise to a 1:1:1 ratio of monounsaturates, polyunsaturates and saturates. The oil later was given to 23 healthy normocholesterolaemic male volunteers who were fed a specially designed whole food diets and compared to palm olein (16:0-rich), canola oil (18:1-rich) or the AHA Step-1 diet, altogether contributing about <200 mg dietary cholesterol/day and 31% energy. Plasma TC and LDL-C were not significantly affected by the three diets regardless of modifications of the primary dietary fatty acids. The high 16:0 palm olein and 18:1 canola brought about nearly identical lipoprotein and plasma cholesterol levels. HDL–C was significantly increased after the AHA diet in comparison to palm olein and canola diets. The results of their dietary fats showed that a balanced fatty acid ratio incorporating palm oil may improve the LDL/HDL-cholesterol ratio and thus this could be cardio-protective.

The Malaysian Palm Oil Board (MPOB) has produced several innovative oil compositions to penetrate niche markets where consumers are concerned with diet and health. Two unique oil compositions with cold stability have been developed from laboratory to pilot plant scale, and are available for commercialization. These oils have palm oil products as base materials and enhanced fatty acid compositions by blending with other vegetable oils. A study of these oils compared with saturated oil or olive oil from the nutrition point of view could help in the promotion of such oils for commercialization and market studies. Therefore, the effects of these novel oils on plasma lipid profiles and their relation to the development of atherosclerotic plaques in a cholesterol-fed rabbit model was explored in the present study.

## 2. Materials and Methods

### 2.1. Materials

Coconut oil (CNO) was obtained from Lee Oil Mills Sdn. Bhd., Malaysia and olive oil was obtained from SOCMA Trading Sdn. Bhd., Malaysia. Both NoveLin I and NoveLin II were obtained from MPOB pilot plant and were produced in accordance with US Patent 7785645 B2 (Process for obtaining an oil composition and the oil composition obtained therefrom) [13].

### 2.2. NoveLin I and NoveLin II Preparation

Two oil compositions, namely NoveLin I and NoveLin II have been developed by the Malaysian Palm Oil Board (MPOB) from laboratory to pilot plant scale (US Patent 7785645 B2). In brief, palm oil is mixed with unsaturated oil such as soybean oil or canola oil. The mixture was heated to a temperature of between 50 °C to 75 °C until all crystals were melted. Then, a fractionation process was carried out at 20 °C, 10 °C and then 8 °C, followed by cooling of the liquid to obtain the oil that resulted in a unique fatty acid composition (Table 1).

### 2.3. Animal Feeding

Animal ethics clearances were approved by the Animal Care and Use Committee of the National University of Malaysia, Kuala Lumpur, Malaysia (FSKB/BIOMED/2010/TILAKAWATI/20-JANUARY/287-JANUARY-2010-JANUARY-2011). Forty New Zealand White rabbits (male) aged five months old and with equal mean body weights were assigned into four experimental groups of ten animals. The rabbits were individually housed in stainless steel cages in an air-conditioned room with temperature controlled at 18 to 23 °C under a 12-h light/12-h dark cycle. All rabbits were fed ad libitum and given free access to water. Experimental rabbits were fed for 100 days on a diet containing a 35% fat energy level with added dietary cholesterol of 0.15% w/w. The fatty acid composition of the dietary oil employed (Table 1) was determined using gas chromatography [6]. The experimental diet groups were classified as follows: (i) NoveLin l, (ii) NoveLin II, (iii) Coconut Oil (CNO) and (iv) Olive Oil (OLV).

During the 100-day feeding period, all animals were handled according to the Standard Guidelines for Use and Care of Laboratory Animals (Ministry of Health Malaysia, 2000). In order to acclimatize the rabbits with the treatment diet, 30% (w/w) of commercial rabbit diet (Cap Persahabatan, FFM Berhad, Selangor, Malaysia) were given during the first week. The commercial pellets, containing approximately 17% crude protein, 2% crude fat, 20% crude fiber, 13% moisture, 0.5% phosphorus, and 0.8–1.5% calcium, were mixed with the treatment diet. Through the second week, the proportion of the commercial rabbit diet was reduced to 15%. During the third and subsequent weeks, rabbits were fed with the treatment diet only. Rabbit weights were measured weekly during the experimental period followed by exsanguination at the end of 100 days via cardiac puncture. Before exsanguination, the rabbits were fasting overnight and anaesthetized with a mixture of ketamine/xylazine (100 mg/mL) and (20 mg/mL) respectively at 0.2 mL/kg body weight. A volume of thirty milliliters of blood was withdrawn, followed by centrifugation at 3000× *g* for 20 min, and kept at −80 °C until further analysis. Several organs of interest including liver, heart, lungs and kidneys were collected and preserved in 10% formalin. The entire aorta was traced, dissected and cleaned off the adherent adventitial tissues, followed by cutting open longitudinally and preservation in 10% formalin prior to staining with oil Red-O for atherosclerotic lesions measurement [14].

### 2.4. Determination of Vitamin E Content

The content of vitamin E in the diets was quantified following AOCS method C-8-89 using High Performance Liquid Chromatography (HPLC) employing a Hewlett Packard HP 1100 system (Hewlett-Packard, Palo Alto, CA, USA) with fluorescence detector (excitation 259 nm, emission 325 nm) and a YMC 150 × 6.0 mm column [15].

### 2.5. Analysis of Plasma Lipid

Blood plasma lipids including TC, TG, LDL–C, HDL–C were analyzed according to manufacturer’s protocols on the Clinical Chemistry Autoanalyser, Roche/Hitachi 902, using enzymatic assay kits (Roche Diagnostics GmbH, Mannheim, Germany).

### 2.6. Measurement of Aortic Lesion Area

After removal of adventitial tissues and fat, the aortas were dissected and opened longitudinally. The aortas were stained in a 1% (w/v) solution of oil red O in 60% (v/v) triethyl phosphate for 20 min and subsequently de-stained with 60% (v/v) triethyl phosphate for 30 min. The stained aortas were photographed and atheromatous plaques were delineated. Plaque area was measured by digital image analysis software (i-SolutionDT, i-MTechnology, Dusan, Daejeon, Korea) and the data was expressed as percentage of the total aortic surface. Lesions were categorized with reference to Idris et al. [14]:

1. Fibrous plaques: Raised nodular lesions, continuous, intense red (when unstained, appear as white hard nodules visible with the naked eye)
2. Fatty plaques: Raised distinct lesions, intensely stained red
3. Fatty streaks: Lipid accumulation, stained light red
4. Lesion free: No plaques or streaks

### 2.7. Statistical Analysis

Data were analyzed using the Statistical Package for Social Science (SPSS, Version 12, IBM Corporation, Armonk, NY, USA)) employing one-way analysis of variance (ANOVA) followed by LSD multiple comparisons (protected LSD) to test differences between dietary treatments. Differences were considered statistically significant at *p* < 0.05.

## 3. Results

### 3.1. Fatty Acid Composition of Diets

With reference to the fatty acid analysis of the diets (Table 1), it was found that the fatty acid ratio of the monounsaturated, polyunsaturated and saturated fatty acids in NoveLin I was very similar to the AHA Step 1 diet, whereas NoveLin II had lower saturated and polyunsaturated fatty acids compared to olive oil (monounsaturated). Olive oil showed the highest content of monounsaturated fatty acids at approximately 76%, with mainly oleic acid. Coconut oil as a control showed the highest content of saturated fatty acids (approximately 92%), consisting of mainly lauric and myristic acids.

### 3.2. Vitamin E Content of Test Diets

The tocopherol and tocotrienol contents of the test diets were also analyzed (Table 2). The OLV diets contained the lowest content of tocopherol (134.20 ppm) compared to the other three diets. In contrast, both palm-based innovative oils contained high contents of tocopherol. The tocopherol contents of NoveLin I and NoveLin II were 357.60 ppm and 251.02 ppm, respectively. Additionally, both NoveLin I and NoveLin II also contained tocotrienols (NoveLin I = 236.61 ppm and NoveLin II = 295.51 ppm, respectively) while CNO and OLV lacked any tocotrienols. The vitamin E contents in NoveLin I and NoveLin II were 594.21 ppm and 546.51 ppm, respectively.

### 3.3. Animal Body and Organ Weights

At the beginning of the study, the rabbits were divided into four groups (NoveLin I, NoveLin II, OLV and CNO) on the basis of equal mean group body weights. Throughout the experimental duration, rabbits fed the experimental diets demonstrated normal growth. Mean body weights of the animals, before and at the end of the study, were not significantly different (*p* > 0.05) between dietary groups following 100 days of dietary intervention (Table 3). The findings indicated that the high-fat atherogenic diet employed in this study did not adversely impact the growth of the animals.

At the time of sacrifice, organ weights—namely heart, lungs, liver, kidneys and spleen—were not significantly different (*p* > 0.05) among all four intervention groups (Table 4).

### 3.4. Lipid Profile of Tested Animals

Table 5 shows TC, TG, LDL–C and HDL–C levels of rabbits in the four dietary groups. The mean plasma TC and LDL–C of the CNO-fed group was the highest and was significantly different when compared to the other three groups. The NoveLin I fed-group had plasma TC and LDL–C concentrations that were slightly higher when compared to both the NoveLin II and OLV-fed groups; however, the difference was not statistically significant. Between the NoveLin II-fed group and OLV-fed group, the TC, LDL–C, TG and HDL–C concentrations were not significantly different from each other. The HDL–C concentration was not statistically significant between all groups (CNO, NoveLin I, NoveLin II and OLV) although the CNO-fed group was observed to have the highest HDL–C concentration. The resulting LDL/HDL–C ratio was highest in the CNO-fed group and statistically significant when compared to the other three groups. In contrast, LDL/HDL–C was significantly lower in the NoveLin II-fed group but attained significance only in comparison to the NoveLin I and CNO-fed groups.

### 3.5. Aortic Lesions Measurement

**Figure 1:**
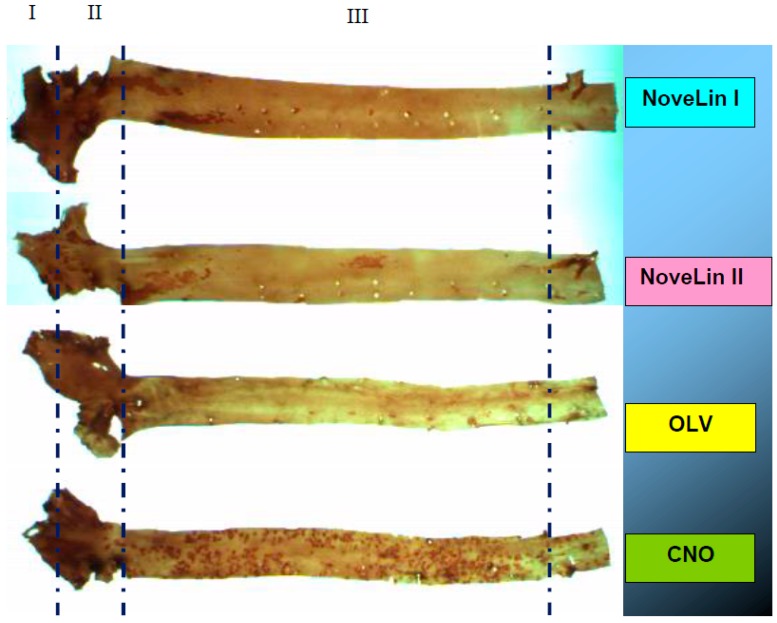
Photographs of aortic lesions in rabbits fed an intervention diet. CNO: Coconut Oil; OLV: Olive Oil. Atherosclerosis development is shown as (I) fibrous plaques, (II) fatty plaques, and (III) fatty streaks.

Following 100 days of feeding with different diet types, all rabbits developed lipid-containing lesions in the intima surface of the aorta. In general, gross pathological examination of the aorta showed abnormal lesions from moderate to high levels of lipid infiltration in the CNO-fed group. Distinct elevations of lesions with white fibrotic caps were evident in several animals in the different dietary groups (Figure 1). The CNO diet resulted in the highest percentage areas of fibrous plaques and fatty plaques compared to the other three groups (NoveLin I, NoveLin II and OLV) (Table 6). The lowest percentage area of fibrous plaques was shown by OLV followed by NoveLin II and NoveLin I, and all were statistically significant compared to CNO. Fatty plaque percentage area was lowest in the NoveLin II group and this was significantly different compared to the other diets. The highest percentage area of fatty streaks was observed in CNO followed by NoveLin I, and this was statistically significant in comparison to the NoveLin II group. The lesion-free area, denoting the total area that was not altered morphologically, was significantly higher in NoveLin I, NoveLin II and OLV groups compared to the CNO group. However, there was no significant difference in lesion-free area between NoveLin I, NoveLin II and OLV groups.

## 4. Discussion

When a new edible oil is introduced, the safety and nutritional qualities of the oil has to be acceptable or above average. The two oils (NoveLin I, NoveLin II) are intended for producing products which are not only marketable in temperate countries, but could be also promoted as healthy oils.

The purpose of the current study was to assess whether the two palm-based innovative oils namely the NoveLin I (which has an approximately equal distribution of saturated, monounsaturated and polyunsaturated fatty acids) and NoveLin II (which has moderately high levels of oleic acids with low content of saturated and polyunsaturated fatty acids), have potent hypocholesterolaemic and anti-atherogenic effects in comparison with olive oil, and whether these properties are connected with changes in plasma lipid compositions in rabbits.

The rabbit is a commonly used experimental model for assessing the effects of dietary fat and development of atherosclerosis. In the current study, a high-fat atherogenic diet formulation containing fat at approximately 35% energy with addition of 0.15% dietary cholesterol was fed to the rabbits. Idris et al. [14] reported that the lesion morphology in rabbits was altered by the percentage of cholesterol added to the diet and the feeding period. Short feeding time with a high percentage of cholesterol (exceeding 2%) may lead to hypercholesterolaemia and atherosclerotic lesions rich in foam cells originated from macrophages. On the other hand, diet supplemented with high-fat, low-dietary cholesterol content, and long feeding duration, may cause atherosclerotic lesions that are rich in smooth muscle cells and contain cholesterol deposits leading to atherosclerotic lesions similar to those of humans [16,17,18].

In the present study, animals were fed a high-fat atherogenic diet daily for 100 days duration. Changes in blood lipids resulting from these fatty acid manipulations were obvious. The test oils used (NoveLin I, NoveLin II, OLV and CNO) have different fatty acid compositions in terms of their saturated:monounsaturated:polyunsaturated ratios. Significant effects were evident in the rabbits fed the CNO diet, which resulted in changes of lipid profiles (TC, LDL–C and TG) that were clearly atherogenic. As a result, the CNO-fed group showed a significantly lower percentage of lesion-free area, with substantial development of fibrous plaques, compared to the other three groups. Similar observations were also reported by Wilson et al. [19] and Kritchevsky et al. [20].

Monounsaturated oils rich in oleic acid are believed to be the healthiest of edible oils in the human diet [11]. Whereas rapeseed, olive and canola oils have in excess of 60% of their fatty acid composition as oleic acid [11], both NoveLin I and NoveLin II, contain about 38% and 53% of oleic acid, respectively. In the present study, both NoveLin oils and olive oil led to significantly lower plasma TC and LDL–C in rabbits compared to the control CNO diet. NoveLin oils are based on palm oil, which has been identified as an exceptional source of two major phytochemicals, including vitamin E (tocopherols and tocotrienols) and carotenoids, both of which are fat-soluble [17]. Vitamin E from palm oil has been reported to be a potent biological antioxidant, protecting against oxidative stress and the atherosclerotic process by modulating pathways of lipid metabolism and fatty acid biosynthesis [21]. It could also decrease the expression of transcription factors regulating adipogenesis and increase apoptosis of adipocytes [21]. A similar finding was reported by Wilson et al. [19] on the effects of emu oil, which is mostly monounsaturated, though less so than olive oil. The same author [19] stated that olive oil, crude emu oil, and refined emu oil diet-fed groups had significantly lower plasma TC and LDL–C concentrations compared to the coconut oil diet-fed group, without differences in plasma HDL–C or TG concentrations. Kritchevsky et al. [20] evaluated the atherogenicity of avocado oil compared to coconut oil, olive oil and corn oil. Their findings showed that serum cholesterol levels were significantly higher in the rabbits fed coconut oil than the other groups, which is commensurate with our findings. Triglyceride levels were similar in all four experimental groups. In addition, Ng et al. [9], who studied dietary effects of palm olein and olive oil in comparison to coconut oil on blood lipids of human subjects, reported that coconut oil demonstrated significantly higher blood lipid parameters i.e., TC, LDL–C and HDL–C; whereas palm olein and olive-fed groups resulted in similar blood lipid parameters.

The literature has highlighted the beneficial effects of polyunsaturated fats and the deleterious impact of saturated fats [22,23]. Currently, monounsaturated fats are regarded as neutral. The atherogenic effects of various oils, such as corn, coconut, olive and avocado, were also evaluated by Kritchevsky et al. [20]. Their findings showed no significant difference in atherogenic effect between avocado oil, a monounsaturated oil, with olive oil, since both contain high levels of oleic acid; however, severe development of aortic lesions was apparent in the coconut oil-fed group, and similar observations were also made in the present study, as rabbits fed coconut oil displayed severe atherosclerosis, most likely due to the high ratio of saturated to monounsaturated and polyunsaturated (S:M:P) fatty acids in their diet [24]. Meanwhile, the severity of lesions in both NoveLin oil groups were very similar to those seen in the olive-fed group, indicating that these newly developed oils have hypocholesterolaemic and anti-atherogenic effects similar to olive oil.

## 5. Conclusions

We found that although the two palm-based innovative oils, namely NoveLin I and NoveLin II, differ in their fatty acid compositions, the dietary effects of these oils on TC, LDL–C and HDL–C were comparable to olive oil in our rabbit study. Besides their fatty acids, the minor components present in both oils, especially the tocotrienols have been reported to reduce TC and LDL–C. Therefore, the outcome of this study indicates that our newly developed oils, especially NoveLin II, have potent hypocholesterolaemic and anti-atherogenic effects that would be beneficial for human health.

## Figures and Tables

**Table 1:** Fatty acid composition of test diets.

Fatty Acid	NoveLin I	NoveLin II	OLV	CNO	Palm Olein [12]	AHA Diet [11]
C8:0				7.51	-	
C10:0				6.27	-	
C12:0				46.42	0.44	
C14:0	0.6	0.66		18.45	1.02	0.2
C16:0	25.97	23.41	11.97	9.45	40.49	25.7
C18:0	3.58	2.82	3.96	2.83	3.99	4.1
C20:0	0.4	0.87	-	0.79	-	
SFA	30.55	27.76	15.93	91.72	45.94	30.0
C18:1	38.22	52.11	76.01	6.76	43.81	37.4
MUFA	38.22	52.11	76.01	6.76	43.81	37.4
C18:2	28.86	17.07	6.78	1.51	10.25	29.3
C18:3	2.37	3.05	0.58	-	-	3.3
PUFA	31.23	20.12	7.36	1.51	10.25	32.6
S:M:P Ratio	1:1:1	0.5:1:0.4	0.2:1:0.1	13.6:1:0.2	1:1:0.2	1:1:1

AHA: American Heart Association; C: Carbon; CNO: Coconut Oil; M: Monounsaturates; MUFA: Monounsaturated Fatty Acid; OLV: Olive Oil; P: Polyunsaturates; PUFA: Polyunsaturated Fatty Acid; S: Saturates; SFA: Saturated Fatty Acid.

**Table 2:** Composition of vitamin E components in test diets (ppm).

Isomers	NoveLin I	NoveLin II	OLV	CNO
alpha-t	94.28	109.96	114.56	163.71
beta-t	10.42	30.88	8.29	0.00
delta-t	64.42	5.87	0.00	12.13
gamma-t	188.48	104.31	11.77	0.00
**Total t**	**357.60**	**251.02**	**134.62**	**175.84**
alpha-t3	59.33	65.12	0.00	0.00
delta-t3	57.11	65.24	0.00	0.00
gamma-t3	120.17	165.15	0.00	0.00
**Total t3**	**236.61**	**295.51**	**0.00**	**0.00**
**Total vitamin E**	**594.21**	**546.51**	**134.62**	**175.84**

CNO: Coconut Oil; OLV: Olive Oil; t = tocopherol; t3 = tocotrienol.

**Table 3:** Animal body weights (g) before and after 100 days of diet intervention.

Body Weights	NoveLin I *n* = 10	NoveLin II *n* = 10	OLV *n* = 10	CNO *n* = 10
Initial (g)	2081.92 ± 107.89 ^a^	2044.92 ± 96.04 ^a^	2016.21 ± 53.17 ^a^	1956.59 ± 69.66 ^a^
Final (g)	2570.00 ± 128.91 ^a^	2550.53 ± 139.41 ^a^	2421.76 ± 156.16 ^a^	2516.84 ± 98.24 ^a^

Values are presented in means ± SD. ^a^ Values within a horizontal row and with similar superscript denote non-significant differences at *p* > 0.05; *n* = number of animals; CNO: Coconut Oil; OLV: Olive Oil.

**Table 4:** Effect of dietary intervention on various organ weights.

Organ Weight (g)	NoveLin I *n* = 10	NoveLin II *n* = 10	OLV *n* = 10	CNO *n* = 10
Heart	4.34 ± 0.20 ^a^	4.24 ± 0.31 ^a^	4.50 ± 0.28 ^a^	4.30 ± 0.34 ^a^
Lungs	11.41 ± 1.31 ^a^	10.26 ± 0.96 ^a^	16.33 ± 1.99 ^a^	13.44 ± 1.62 ^a^
Liver	55.26 ± 4.65 ^a^	55.86 ± 4.27 ^a^	56.78 ± 5.24 ^a^	52.58 ± 4.35 ^a^
Kidneys	13.63 ± 0.83 ^a^	12.54 ± 1.05 ^a^	13.02 ± 0.58 ^a^	13.33 ± 0.75 ^a^
Spleen	0.62 ± 0.10 ^a^	0.54 ± 0.07 ^a^	0.69 ± 0.05 ^a^	0.45 ± 0.05 ^a^

Values are presented as means ± SD. ^a^ Values within a horizontal row and with similar superscript denote non-significant differences at *p* > 0.05; *n* = number of animals; CNO: Coconut Oil; OLV: Olive Oil.

**Table 5:** Effect of dietary intervention on plasma lipid profiles of rabbits.

Plasma Lipid (mmol/L)	NoveLin I *n* = 10	NoveLin II *n* = 10	OLV *n* = 10	CNO *n* = 10
TC	15.53 ± 2.84 ^a^	13.93 ± 2.49 ^a^	13.16 ± 2.14 ^a^	18.67 ± 2.36 ^b^
TG	2.29 ± 1.22	1.61 ± 1.02	2.03 ± 0.98	2.18 ± 0.47
LDL–C	13.15 ± 2.54 ^a^	12.14 ± 2.21 ^a^	12.74 ± 1.98 ^a^	16.60 ± 2.08 ^b^
HDL–C	1.86 ± 0.29	1.67 ± 0.17	1.77 ± 0.25	2.11 ± 0.15
LDL/HDL	5.08 ± 1.63 ^a^	3.91 ± 1.65 ^b^	4.22 ± 2.01 ^a,b^	8.43 ± 2.10 ^c^

Values are presented as means ± SD. ^a–c^ Values within a horizontal row and with different alphabetic letters denote significant differences at *p* < 0.05; *n* = number of animals; CNO: Coconut Oil; OLV: Olive Oil.

**Table 6:** Percentage of fibrous plaques, fatty plaques, fatty streaks and lesion free areas in the aorta of rabbits fed intervention diets.

Diet	Fibrous Plaque	Fatty Plaque	Fatty Streak	Free Lesion
NoveLin I	1.57 ± 0.77 ^b^	3.68 ± 1.57 ^b^	8.37 ± 1.89 ^a,b^	86.3 ± 3.92 ^b^
NoveLin II	0.64 ± 0.40 ^c^	1.47 ± 0.66 ^c^	3.87 ± 1.32 ^c^	93.86 ± 6.49 ^b^
OLV	0.30 ± 0.15 ^d^	3.51 ± 1.92 ^b^	7.06 ± 1.98 ^b^	88.95 ± 1.55 ^b^
CNO	6.45 ± 3.36 ^a^	5.78 ± 1.84 ^b^	9.99 ± 1.56 ^a^	77.47 ± 2.28 ^a^

Values are presented as means ± SD. ^a–d^ Values within a horizontal row and with different alphabet letters denote significant differences at *p* < 0.05; CNO: Coconut Oil; OLV: Olive Oil.
